# Lung function is related to salivary cytokines and hormones in healthy children. An exploratory cross‐sectional study

**DOI:** 10.14814/phy2.15861

**Published:** 2023-12-12

**Authors:** Laura Gochicoa‐Rangel, Jaime Chávez, Rodrigo Del‐Río‐Hidalgo, Selene Guerrero‐Zúñiga, Uri Mora‐Romero, Rosaura Benítez‐Pérez, Luis Rodríguez‐Moreno, Luis Torre‐Bouscoulet, Mario H. Vargas

**Affiliations:** ^1^ Departamento de Fisiología Respiratoria Instituto Nacional de Enfermedades Respiratorias Ismael Cosío Villegas Mexico City Mexico; ^2^ Instituto de Desarrollo e Innovación en Fisiología Respiratoria Mexico City Mexico; ^3^ Departamento de Investigación en Hiperreactividad Bronquial Instituto Nacional de Enfermedades Respiratorias Ismael Cosío Villegas Mexico City Mexico; ^4^ Present address: Servicio de Pediatría Nuevo Hospital Civil Guadalajara Mexico; ^5^ Present address: Servicio de Neumología Pediátrica Hospital Puebla Puebla Mexico

**Keywords:** diffusing capacity for carbon monoxide, glucagon, GM‐CSF, granulocyte‐macrophage colony‐stimulating factor, lung function, oscillometry, pulmonary function tests, spirometry

## Abstract

Pulmonary mechanics has been traditionally viewed as determined by lung size and physical factors such as frictional forces and tissue viscoelastic properties, but few information exists regarding potential influences of cytokines and hormones on lung function. Concentrations of 28 cytokines and hormones were measured in saliva from clinically healthy scholar children, purposely selected to include a wide range of body mass index (BMI). Lung function was assessed by impulse oscillometry, spirometry, and diffusing capacity for carbon monoxide, and expressed as z‐score or percent predicted. Ninety‐six scholar children (55.2% female) were enrolled. Bivariate analysis showed that almost all lung function variables correlated with one or more cytokine or hormone, mainly in boys, but only some of them remained statistically significant in the multiple regression analyses. Thus, after adjusting by height, age, and BMI, salivary concentrations of granulocyte‐macrophage colony‐stimulating factor (GM‐CSF) in boys were associated with zR5‐R20 and reactance parameters (zX20, zFres, and zAX), while glucagon inversely correlated with resistances (zR5 and zR20). Thus, in physiological conditions, part of the mechanics of breathing might be influenced by some cytokines and hormones, including glucagon and GM‐CSF. This endogenous influence is a novel concept that warrants in‐depth characterization.

## Graphical abstract

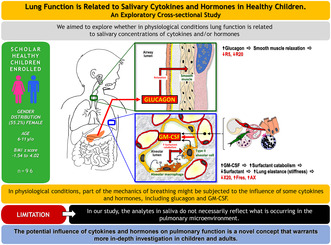

## 1 INTRODUCTION

Pulmonary function in healthy subjects, particularly with respect to lung mechanics, has been traditionally viewed as mainly determined by lung size (Cook et al., 1958) and by well‐known mechanical factors such as respiratory muscle‐driven forces, frictional forces opposing air movement, viscoelastic properties of lung parenchyma, and thoracic cage, etc. (Kaminsky & Irvin, 2018; Quanjer et al., 1993). In this context, raw data obtained through pulmonary function tests (PFTs) greatly depend on the subject's characteristics such as height, sex, age, and weight. Thus, PFTs results need to be interpreted after their adjustment by these factors using reference equations obtained in a large group of healthy people. Nevertheless, even after this adjustment a certain amount of within‐ and between‐subject variability is usually found in PFTs. Up to now, this “biological variability” remains largely unexplained and suggests that some additional influential factors might be still present.

A lot of endogenous mediators in the body have pleiotropic effects, which means that they not only act on their major target tissues but also on other secondary cells and tissues. Therefore, it is possible that lung function might be also influenced by mediators such as cytokines and hormones. However, as far as we know, aside from few hormones/neurotransmitters such as catecholamines, acetylcholine, and histamine (Barnes, 1985), this assumption has been seldom explored. An example of such mediators with pleiotropic effects is the pancreatic hormone glucagon, which beyond its preeminent role in glucose metabolism also has a bronchodilator effect (Sherman et al., 1988; Wilson & Nelson, 1990).

In the present study, we aimed to investigate whether cytokines and hormones measured in saliva may account for the between‐subject variability of lung function, as assessed by impulse oscillometry (IOS), spirometry, and diffusing capacity for carbon monoxide (DL_CO_), in a population purposely selected to include children with a wide range of body mass index (BMI). In view of the exploratory nature of the study and envisaging a potential link of lung function with adipose tissue, we tried to include cytokines and hormones involved in the metabolism of fat tissue. Saliva contains many chemical substances and is increasingly used as biological fluid because it can be sampled noninvasively, and its analyte concentrations may provide useful information of biological relevance (Amado et al., 2019; Chávez‐Alderete et al., 2021; Nunes et al., 2015).

## 2 METHODS

### 2.1 Population and study design

This was a prospective, cross‐sectional study of children recruited from primary schools located near Mexico City. The protocol was approved by the institutional review board from the Instituto Nacional de Enfermedades Respiratorias (approval number C07‐14) and procedures were carried out in accordance with The Code of Ethics of the World Medical Association (Declaration of Helsinki). After obtaining the permission from scholastic authorities, a brief letter explaining the objectives and methods of the study was sent to the children's parents. Those who agreed with the participation of their son or daughter in the study were asked to fill a questionnaire inquiring the child's clinical information and to sign a formal consent letter. We also requested the child consent in an assent letter. Children with habitual passive tobacco smoke exposure, with data suggestive of chronic lung diseases, gastroesophageal reflux, or periodontal, cardiologic, renal, hepatic, neuromuscular, rheumatologic, or with acute infectious diseases were excluded. Children unable to adequately perform the PFTs procedures were eliminated from the study. Sample size was determined by feasibility.

On the day of the study, a physical examination was done, including measurements of height (model 206 scale, Seca GMBH & Co., Hamburg, Germany) and weight (model UM‐061 scale, Tanita UK Ltd. Middlesex, United Kingdom), and from these measurements the z‐score of body mass index for age (zBMI) was calculated according to WHO charts. Afterward, a saliva sample was collected and PFTs were performed in the following order: IOS, DL_CO_, and spirometry. To minimize the influence of circadian rhythms, saliva sampling, and PFTs were always done between 10 am and 1 pm.

### 2.2 IOS

IOS is based on transient modifications of tidal breathing caused by brief sounds, each one composed by sinusoidal waves at frequencies from ~5 to ~20 Hz. Change in the pressure/flow ratio yields an estimate of the respiratory system impedance, which is composed by resistance (R), mainly reflecting opposition to airflow, and reactance (X), mainly reflecting elastic and inertial forces. Because higher frequency sound waves do not reach distal airways, values of resistance at 20 Hz (R20) reflect the status of large airways, while values at 5 Hz (R5) indicates the status of the entire respiratory system, and their difference (zR5‐R20) reflects peripheral airways resistance.

IOS was done using a Jaeger MS‐IOS (CareFusion, Germany), calibrated daily for volume and weekly for air flow, with the child seated, wearing a nose clip, with cheeks supported by the hands of a researcher, and breathing quietly at tidal volume through an antimicrobial filter. Three 30‐s measurements that fulfilled acceptability and repeatability criteria were obtained, and their average was considered the final value.

### 2.3 DL_CO_

Measurement of DL_CO_ was done in an EasyOnePro system (ndd Medical Technologies, Zurich, Switzerland) according to the 2005 ATS/ERS statement (MacIntyre et al., 2005). Once the mouthpiece and nose clip were in place, the child performed some breaths at tidal volume. When a stable functional residual capacity (FRC) was achieved, the child made an unforced exhalation down to residual volume, followed by one quick inhalation of the test gas (0.3% CO, 10% He, 21% O_2_, in N_2_) up to total lung capacity, which was sustained during 8–12 s, and followed by a smooth and unforced exhalation, without hesitations or interruptions. This maneuver was repeated up to five times, 4 min apart, until at least two acceptable maneuvers were obtained. The final value was expressed as the average of the two acceptable maneuvers.

### 2.4 Spirometry

Forced spirometry was done with an EasyOne on PC system (ndd) and following the 2005 ATS/ERS recommendations (Miller et al., 2005). After explanation and demonstration of the maneuver, and with a mouthpiece and nose clip in place, the child performed a deep inspiration followed by a forceful exhalation until a plateau was achieved. This maneuver was repeated until three acceptable and repeatable maneuvers were obtained.

### 2.5 Saliva sampling and processing

Procedures for the saliva sampling and processing were detailed elsewhere (Chávez‐Alderete et al., 2021). Saliva samples were obtained after at least 2 h of fasting. Once advanced caries or evident gingival disease were discarded, a 10‐s mouthwash with water was carried out and at least 5 mL of unstimulated, whole saliva sample was collected by passive drooling. The sample was immediately centrifuged at 4000 rpm, and the supernatant was aliquoted and maintained at 4°C, and before 12 h put at −70°C. On the day of the assay, salivary analytes were measured by multiplex with a Luminex Bio‐Plex 200 (Bio‐Rad, Austin, TX, USA). Analytes included were interleukin (IL)‐1β, IL‐2, IL‐4, IL‐5, IL‐6, IL‐7, IL‐8, IL‐10, IL‐12, IL‐13, IL‐17, granulocyte colony stimulating factor (G‐CSF), granulocyte‐macrophage colony‐stimulating factor (GM‐CSF), interferon gamma (IFN‐γ), monocyte chemoattractant protein 1 (MCP‐1), macrophage inflammatory protein 1β (MIP1β), and tumor necrosis factor alfa (TNF‐α) (Bio‐Plex Pro Human Cytokine Grp I Panel 17‐Plex, Cat. #M50‐00031YV, Bio‐Rad), as well as ghrelin, glucose‐dependent insulinotropic polypeptide (GIP), glucagon‐like peptide 1 (GLP‐1), glucagon, insulin, leptin, plasminogen activator inhibitor 1 (PAI‐1), resistin, visfatin, C peptide (Bio‐Plex Pro Human Diabetes Panel 10‐Plex, Cat. #171‐A7001M, Bio‐Rad), and adiponectin (Bio‐Plex Pro Human Diabetes Adiponectin Assay, Cat. #171‐A7003M, Bio‐Rad). Values below the detection limit were considered as to be 0.1 pg/mL lower than the lowest concentration detected for that analyte. Dilution of saliva was adjusted by dividing the multiplex results by the albumin concentration measured by the Bradford microassay (Protein Assay, Cat. #500‐0006, Bio‐Rad).

### 2.6 Data analysis

Because noticeable differences between male and female children are usually observe for PFTs variables, and because our preliminary analyses showed that associations of cytokines/hormones with lung function differed between boys and girls, all analyses were performed separately for each sex. Each PFTs variable was evaluated as raw values or as values converted to their respective z‐scores (z) according to reference populations (Gochicoa‐Rangel et al., 2015, 2019; Martínez‐Briseño et al., 2020). Normal distribution of all variables was corroborated through the Kolmogorov–Smirnov test (the original concentration of salivary biomarkers was first log‐transformed to achieve a normal distribution). Data in text and illustrations were expressed as mean ± standard deviation. Comparisons were assessed by the non‐paired Student's *t*‐test, and bivariate associations were evaluated with the Pearson correlation.

Because of the large influence of age, height, and BMI on PFTs, the potential association of salivary analytes with the lung function variables was always assessed by adjusting for these parameters. In this context, for the multivariable analysis two approaches were explored.

- *Model 1*: Multiple linear regressions with the raw values of each PFTs parameter (dependent variable) versus age, height, zBMI, and the analyte being evaluated (independent variables). In order to assess generalizability of the results, internal validation was performed on each Model 1 regression through the bootstrapping method (500 resamples) for assessing its discrimination (optimism‐adjusted R^2^ and g‐index) and calibration (optimism‐adjusted slope) capabilities (Harrell Jr., 2015; Steyerberg, 2019).
- *Model 2*: Multiple linear regressions with the z‐scores of each PFTs parameter (dependent variable) versus age, height, zBMI, and all salivary analytes that in the bivariate analysis were associated with that PFTs variable (independent variables).

Statistical analyses were performed using the R program (*rms* package for internal validation) and Stata v13. Statistical significance was set at one‐tailed *p* < 0.05.

## 3 RESULTS

The study included 96 clinically healthy children (55.2% females), aged between 6.0 and 11.9 years, with BMI z‐scores varying from −1.54 to 4.02. Characteristics of the study population and results of PFTs can be observed in Table 1. No statistically significant differences between males and females were found except for zFres, which was higher in females as compared with males (*p* = 0.03).

**TABLE 1:** Characteristics of the study population and results from pulmonary function tests.

Variable	All (*n* = 96)	Males (*n* = 43)	Females (*n* = 53)	p^a^
Birth weight (kg)	3.28 ± 0.04 (2.50, 4.20)	3.26 ± 0.06 (2.60, 4.20)	3.30 ± 0.06 (2.50, 4.05)	0.36
Age (years)	9.5 ± 1.8 (6.0, 11.9)	9.6 ± 1.9 (6.0, 11.9)	9.4 ± 1.8 (6.0, 11.8)	0.62
Weight (kg)	36.1 ± 11.0 (16.8, 65.9)	35.1 ± 10.0 (16.8, 61.5)	36.9 ± 11.8 (17.1, 65.9)	0.44
Height (cm)	135 ± 12 (109, 161)	134 ± 11 (109, 152)	136 ± 12 (111, 161)	0.34
BMI (kg/m^2^)	19.3 ± 3.6 (13.4, 27.7)	19.3 ± 3.6 (13.8, 27.7)	19.4 ± 3.7 (13.4, 27.2)	0.84
zBMI	1.02 ± 1.24 (−1.54, 4.02)	1.06 ± 1.3 (−1.42, 4.02)	0.98 ± 1.19 (−1.54, 3.73)	0.74
zR5	0.33 ± 0.80 (−1.31, 2.25)	0.27 ± 0.80 (−1.23, 2.23)	0.38 ± 0.80 (−1.31, 2.25)	0.52
zR20	0.39 ± 0.85 (−2.45, 2.44)	0.34 ± 0.82 (−1.38, 2.44)	0.43 ± 0.88 (−2.45, 1.96)	0.63
zR5‐R20	−0.01 ± 0.94 (−2.85, 4.80)	−0.01 ± 1.03 (−2.85, 4.80)	0 ± 0.83 (−1.50, 2.30)	0.99
zX5	−0.32 ± 0.73 (−2.34, 1.60)	−0.29 ± 0.68 (−1.99, 1.60)	−0.35 ± 0.78 (−2.34, 1.08)	0.69
zX20	−0.29 ± 0.82 (−2.57, 2.62)	−0.15 ± 0.77 (−1.55, 1.59)	−0.40 ± 0.84 (−2.57, 2.62)	0.13
zFres	0.29 ± 1.02 (−2.35, 2.33)	0.03 ± 0.95 (−2.35, 1.52)	0.50 ± 1.03 (−2.20, 2.33)	**0.03**
zAX	0.23 ± 0.76 (−1.48, 3.33)	0.10 ± 0.68 (−1.48, 1.71)	0.34 ± 0.81 (−0.80, 3.33)	0.13
zFEV_1_	−1.06 ± 1.80 (−6.78, 2.71)	−0.78 ± 1.56 (−3.88, 2.71)	−1.29 ± 1.96 (−6.78, 1.70)	0.17
zFVC	−0.97 ± 1.95 (−6.68, 4.56)	−0.62 ± 1.84 (−4.37, 4.56)	−1.26 ± 2.01 (−6.68, 2.54)	0.11
zFEV_1_/FVC	−0.26 ± 1.01 (−3.32, 2.12)	−0.26 ± 0.84 (−1.94, 2.12)	−0.27 ± 1.14 (−3.32, 2.04)	0.98
zDL_CO_	−0.21 ± 1.09 (−4.25, 1.85)	−0.14 ± 0.99 (−2.87, 1.67)	−0.26 ± 1.16 (−4.25, 1.85)	0.58

^a^ Student's *t*‐test. All variables from pulmonary function tests and BMI were transformed to their respective z‐scores according to predicted values.

*Note*: Data correspond to mean ± standard deviation (minimum, maximum).

Abbreviations: AX, reactance area; BMI, body mass index; DL_CO_, lung diffusion for carbon monoxide; FEV_1,_ forced expiratory volume at first second; Fres, resonance frequency; FVC, forced vital capacity; R, resistances at 5 and 20 Hz; X, reactances at 5 and 20 Hz.

The raw and albumin‐adjusted concentrations of all salivary analytes derived from this population were recently published elsewhere (Chávez‐Alderete et al., 2021). IL‐2 and IL‐5 were not detected in a high number of samples (91% and 75%, respectively), so these cytokines were excluded from subsequent analyses.

There were no statistically significant differences in concentrations of salivary analytes between boys and girls (data not shown).

Almost all PFTs variables had some positive or negative bivariate correlation with one or more salivary analytes, but in general terms, such associations were more frequently observed in males than in females (Table 2). In the multiple linear regression analyses, only some of these associations remained significant after their adjustment by age, height, and zBMI, regardless of whether PFTs raw values (Model 1) or z‐scores (Model 2) were used. As shown in Tables S1 and S2, after assessing the internal validation of associations observed in Model 1, we found that discrimination (*R* ^2^, g‐index) and calibration (slope) indexes maintained a good performance after their adjustment by the level of optimism, indicating good reproducibility of results.

**TABLE 2:** Bivariate Pearson rho coefficients of salivary analytes associated (*p* < 0.05) with z‐scores of pulmonary function tests (PFTs) in 96 scholars, and their inclusion in two models of multiple linear regression (MLR), both adjusted by age, height, and zBMI.

Variable	zR5	zR20	zR5‐R20	zX5	zX20	zFres	zAX	zFEV_1_	zFVC	zFEV_1_/FVC	zDL_CO_
Males (*n* = 43)											
IL‐7	—	—	—	—	—	—	—	—	—	—	0.33^a^
IL‐8	—	—	—	—	—	0.24^a^	—	—	—	**0.29** ^a^ ^,^ ^b^	—
IL‐10	—	—	—	—	−0.26	0.28	—	—	—	—	—
IL‐13	−0.25	—	−0.22	—	—	—	—	—	—	—	—
GM‐CSF	—	—	**0.37** ^a^ ^,^ ^b^	—	**−0.43** ^a^ ^,^ ^b^	**0.43** ^a^ ^,^ ^b^	**0.36** ^a^ ^,^ ^b^	0.06^a^	—	—	—
MIP1ß	—	—	—	—	—	—	—	—	—	0.27	**0.34** ^b^
C Peptide	—	—	—	—	—	0.30	—	—	−0.30	—	—
Ghrelin	—	—	—	—	—	—	—	−0.31	−0.36	—	—
GIP	—	—	—	—	—	—	—	—	−0.27	0.26	—
GLP‐1	−0.30^a^	−0.30	—	—	—	—	—	—	−0.29	0.28	—
Glucagon	**−0.34** ^a^ ^,^ ^b^	**−0.36** ^a^ ^,^ ^b^	—	—	—	—	—	—	−0.33	0.28	—
Leptin	—	—	−0.20	—	—	—	—	—	**−0.42** ^b^	0.31	—
PAI1	—	—	—	—	—	0.27	—	—	—	—	—
Visfatin	—	—	—	—	—	0.27	—	—	—	—	—
Adiponectin	—	—	—	—	—	—	—	—	—	0.11^a^	—
Females (*n* = 53)											
IL‐1ß	—	—	—	—	—	—	—	—	0.24	—	—
IL‐7	—	—	—	—	—	—	0.33	—	—	—	—
IL‐10	—	—	—	—	−0.29	—	**0.34** ^a^ ^,^ ^b^	—	—	—	—
IL‐12	—	—	**0.27** ^b^	—	—	—	0.30	—	0.24	—	—
IL‐17	—	—	—	—	—	—	—	—	—	−0.24	—
GM‐CSF	—	—	—	—	—	—	—	—	0.24	—	—
MCP1	—	—	—	—	—	—	—	—	0.24	—	—
C peptide	—	−0.24	—	—	—	—	—	—	**0.30** ^b^	—	—
Ghrelin	—	—	—	—	—	—	—	—	0.23	—	—
GIP	—	—	—	—	—	0.26	—	—	—	—	—
Glucagon	—	—	—	—	—	—	—	—	0.26	—	—
Insulin	—	−0.23^a^	0.16^a^	—	—	—	—	—	—	—	—
Leptin	—	—	—	—	—	—	—	—	0.24	—	—

^a^ Analyte included in the final Model 1, that is, MLR with the raw value of each PFTs parameter (dependent variable) versus age, height, zBMI, and the analyte being evaluated (independent variables).

^b^ (Bold values) = Analyte included in the final Model 2, that is, MLR with the z‐score of each PFTs parameter (dependent variable) versus age, height, zBMI, and all analytes with statistical significance in the bivariate Pearson correlation (independent variables). The scatterplot can be observed in Figure 1.

*Note*: Raw values of analytes were always adjusted by albumin and log‐transformed (log[pg/μg albumin]). For definition of abbreviations, see the main text.

**FIGURE 1:**
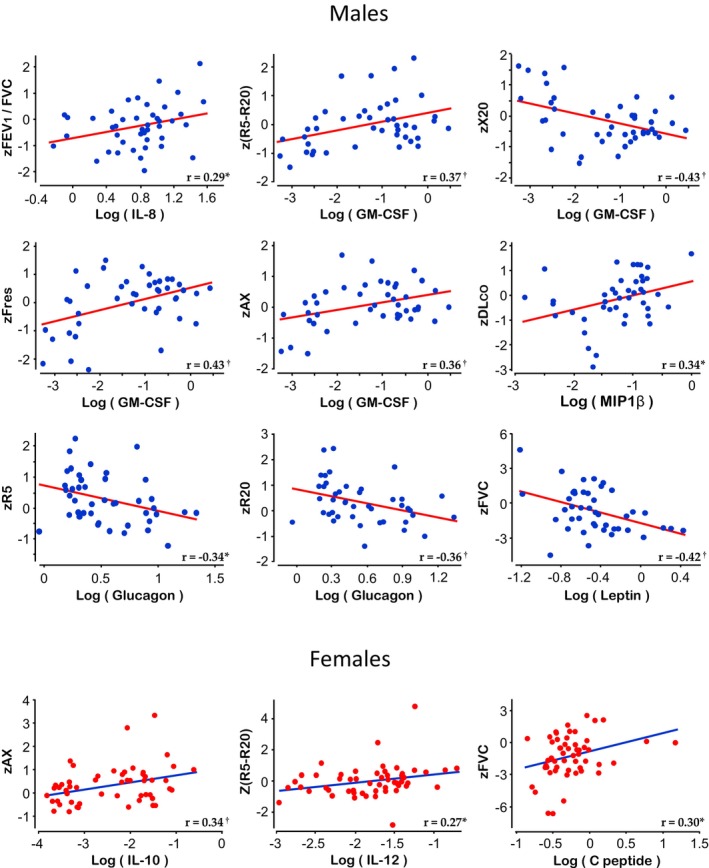
Correlations between lung function and salivary cytokines or hormones in clinically healthy children. Data in x‐axes correspond to albumin‐adjusted salivary concentrations in log[pg/μg albumin], and in y‐axes to the z‐score (z) of lung function. AX, reactance area; DL_CO,_ lung diffusion for carbon monoxide; FEV_1,_ forced expiratory volume at first second; Fres, resonance frequency; FVC, forced vital capacity; GM‐CSF, granulocyte‐macrophage colony‐stimulating factor; IL, interleukin; MIP, macrophage inflammatory protein; R5 and R20, resistances at 5 and 20 Hz; X20, reactance at 20 Hz. Insets correspond to the Pearson rho coefficient and its statistical significance (*p < 0.05; ^†^ *p* < 0.01).

Among the two models of multiple linear regressions, Model 2 is more informative than Model 1 because each analyte is not only adjusted by age, height, and zBMI but also by other analytes potentially involved in the prediction of pulmonary function. In this context, the most notable associations remaining in Model 2 were as follows (Table 2). In boys, GM‐CSF was associated with zR5‐R20 and reactance parameters (zX20, zFres, and zAX), and glucagon inversely correlated with resistance parameters (zR5 and zR20). Although four hormones (GIP, GLP‐1, glucagon, and leptin) had an inverse association with zFVC in the bivariate analysis, which might partly explain the positive association of these hormones with the zFEV_1_/FVC ratio, in the multivariable approach only leptin remained associated with zFVC. Other associations remaining statistically significant in the multivariable analyses were zFEV_1_/FVC with IL‐8, and zDL_CO_ with MIP1β. By contrast, in girls, fewer associations persisted in Model 2, such as zR5‐R20 with IL‐12, zAX with IL‐10, and zFVC with C peptide. Figure 1 shows the bivariate scatterplots of all independent variables that retained statistical significance in the multivariable analyses and were included in the final Model 2, while Figure S1 illustrates how standardized beta coefficients change when the raw values of lung function are associated with the cytokine/hormone, with or without inclusion of age, height, and zBMI as covariables.

## 4 DISCUSSION

Our results suggest that in healthy children, mechanical features of lung function are partly influenced by cytokines and hormones. As far as we know, this is a novel concept pointing out that pulmonary mechanics are not only determined by lung size or physical properties of airways and lung parenchyma, but that after adjusting by the strong influence of height, age, and BMI, several bioactive molecules are still able to produce subtle changes in lung function.

We found that salivary concentration of glucagon had a significant inverse correlation with IOS resistances in males. Glucagon is a hormone released by pancreatic α‐cells that regulates insulin secretion and exerts a biological effect on several organs, including liver, fat tissue, heart, and skeletal and smooth muscles. It has been described that glucagon has a relaxing effect on several smooth muscles, including esophageal, intestinal, vascular, and airway smooth muscles (Insuela et al., 2015; Lin & Zhang, 1989). The relaxing effect of glucagon might be due to either direct stimulation of glucagon receptors in smooth muscle (Santamaria et al., 1991), or indirectly through the epithelium‐dependent release of PGE_2_ and NO (Insuela et al., 2015). Interestingly, glucagon receptors are G_αs_ protein‐coupled receptors that after their activation lead to intracellular cAMP increase, which is the same signaling pathway triggered by β_2_‐adrenoceptor‐stimulating drugs, the most used bronchodilators. Thus, in our study, the decreasing trend of respiratory resistances with increasing levels of glucagon in boys might be explained by these smooth muscle relaxing mechanisms. A more intriguing finding was the negative association of salivary glucagon with zFVC. It is known that the ultimate factor limiting the amount of air forcefully exhaled by the subject, and thus determining FVC, is the functional closure of small airways (Irvin & Wanger, 2018). These small airways are maintained opened due to pulmonary surfactant and retractile forces generated by the transpulmonary pressure (R_L_). Therefore, a decrease of R_L_ should favors a premature closure of small airways (Heil et al., 2008). In this context, it might be possible that glucagon caused relaxation of contractile elements inside the lung parenchyma interstitium, such as vascular smooth muscle (Farah, 1983) and myofibroblasts, thus reducing R_L_ and promoting premature small airway closure and FVC limitation. It might be argue that such an effect should have impact IOS parameters putatively reflecting small airways (R5‐R20, X5, Fres, and AX) (Cottini et al., 2021), which was not the case (Table 2). Because fluctuations of transpulmonary pressure during tidal breathing is much lower than during a forceful exhalation, it might be possible that the glucagon‐induced loss of tethering forces was not so intense as to produce small airway closure during IOS, but high enough as to favor it during spirometry. However, all these speculations awaits to be demonstrated.

Contrasting with the possible role of glucagon on respiratory resistances, salivary levels of GM‐CSF were mainly associated with respiratory reactances, though this was true only for males. Thus, the higher the GM‐CSF levels, the lower the X20 values and, correspondingly, the higher the Fres and the AX values, which may imply that GM‐CSF produced an increase in lung elastance. GM‐CSF into the alveolus mainly comes from alveolar epithelial cells, but other extra‐alveolar sources of GM‐CSF may be lymphocytes, granulocytes, endothelial cells, fibroblasts, etc (Mir‐Kasimov et al., 2012; Shiomi & Usui, 2015). In the mid 1990‐decade, it was shown that GM‐CSF knockout mice developed pathological lesions similar to pulmonary alveolar proteinosis, a disease characterized by accumulation of alveolar surfactant lipoproteins (Stanley et al., 1994). This finding suddenly unveiled a major role played by GM‐CSF on surfactant homeostasis (Reed & Whitsett, 1998), leading shortly thereafter to the discovery that human pulmonary alveolar proteinosis is due to impairment of the GM‐CSF signaling (Dirksen et al., 1997). Currently, exogenous administration of GM‐CSF has become a therapeutical option for alveolar proteinosis. Alveolar macrophages and type II pneumocytes, along with other cell types, express the GM‐CSF receptor, and stimulation of this receptor enhances the catabolism of surfactant, especially by alveolar macrophages (Trapnell & Whitsett, 2002). Therefore, a potential mechanism linking the increasing levels of GM‐CSF with lower X20 values and higher Fres and AX is that GM‐CSF decreased the amount of alveolar surfactant (through its accelerated catabolism) with a secondary increase of surface tension and higher lung elastance (Suki et al., 2011). On the other hand, a high surface tension not only promotes alveolar collapse but also favors the small airways closure, which might be the reason why zR5‐R20 augmented as salivary GM‐CSF increased.

Leptin is a hormone mainly released by adipocytes, but also by other cells. It is involved in satiety perception with an appetite suppressing effect. Our results showed that, in boys, salivary levels of leptin inversely correlated with FVC. This finding is in line with some studies in adults that described an inverse correlation between plasma or serum concentration of leptin and either FVC or FEV_1_ (Garshick et al., 2018). The mechanism of this inverse association is not clear. Although at first sight this effect might be partly explained by the association of leptin with obesity, the leptin receptor is widely expressed in many tissues, including the lung (Bakshi et al., 2021), and thus a direct effect of leptin on pulmonary structures cannot be discarded.

Some other associations between salivary proteins and lung function were also present, but for now their relevance and potential mechanisms are still uncertain. This was the case for IL‐8 with zFEV_1_/FVC, and MIP1β with zDL_CO_ in boys, and IL‐10 with zAX, IL‐12 with zR5‐R20, and C peptide with zFVC in girls.

Our results showed a striking difference between males and females regarding associations of PFTs and salivary analytes, inasmuch as almost all associations were observed in males. An explanation for these sex‐related differences is difficult to envisage. Sex hormones may influence lung function, either on lung mechanics by androgens (Montano et al., 2020) or on lung immunity, inflammation, and vascular reactivity by estrogens and progesterone (Tam et al., 2011). Nevertheless, our population was integrated by children between 6 and 11.9 years, a life period when levels of sex hormones are similar in both genders, perhaps except for oldest children who are close to puberty (Elmlinger et al., 2005). A more plausible explanation might be that these sex‐related differences are due to a dysanaptic growth of airways and lungs in boys, with a slower growth of the larger airways than lung volume (Pagtakhan et al., 1984). Finally, it has been described small but statistically significant differences between boys and girls regarding blood concentrations of trace elements such as Zn, Fe, and Cu (Cao et al., 2016), and at least for Zn a positive association with lung function (FVC and FEV_1_) has been reported (Pan et al., 2020).

Our study has some potential limitations. (1) Analytes in saliva not necessarily reflects what is occurring in the pulmonary microenvironment. Although this assertion may be true, there is an increasing number of studies pointing out that salivary concentrations of analytes correlate not only with respiratory diseases but even in neurological, metabolic/endocrine, and inflammatory/autoimmune diseases (Tvarijonaviciute et al., 2020). For example, salivary concentration of LTE_4_ was higher in adults with aspirin‐intolerant asthma, as compared with those with aspirin‐tolerant asthma (Gaber et al., 2008). A research group in South Africa reported that increased levels of some biomolecules in saliva, mainly C‐reactive protein, serum amyloid P, ferritin, and MCP‐1, have diagnostic capabilities in pulmonary tuberculosis (Jacobs et al., 2016; Phalane et al., 2013). Likewise, increased salivary C‐reactive protein, at a cut off value of 3.8 ng/L, had good sensitivity (91.4%) and specificity (80.9%) to diagnose neonatal pneumonia (Omran et al., 2018). Finally, C‐reactive protein, procalcitonin, and neutrophil elastase in saliva were higher in COPD patients, correlated with symptoms, and had a further increase during exacerbation of the disease (Patel et al., 2015). Thus, as salivary analytes may reflect the pulmonary condition in the diseased state, it is conceivable that they might be also associated with lung function in the healthy state. (2) Similar to their serum counterparts (Coskun et al., 2023), salivary analytes might vary according to many external/internal conditions or circadian/ultradian rhythms, and we did not controlled for such factors. However, it is known that lung function is subjected to such variability too and its circadian rhythmicity has been well characterized (Spengler & Shea, 2000). Thus, although at first glance the variability of cytokines and hormones could be seen as a problem, in fact it is advantageous because if this variability matches the variability of lung function, then a potential causal role can be speculated. For example, part of the known circadian variability of lung function might be related to changes in cytokines or hormones, which is supported by some studies showing that during the early morning (a time coinciding with the lowest lung function), serum concentration of GM‐CSF is higher (Dincol et al., 2000), while plasma concentration of glucagon is lower (Behrman et al., 1978; Jauch‐Chara et al., 2007). (3) Our study was observational and cross‐sectional in nature and the associations that we found do not necessarily imply cause‐effect relationships. (4) Our study was exploratory in nature, and although according to the internal validation our results had good reproducibility, the study must be viewed as a hypothesis‐generating study and our conclusions must be validated by further studies.

## 5 CONCLUSIONS

Lung function in healthy subjects has been traditionally viewed as mainly determined by physical factors such as lung size, frictional forces, and viscoelastic properties of tissues. However, our results pointed out that part of the mechanics or breathing might be subjected to influence of some cytokines and hormones, mainly glucagon and GM‐CSF, in physiological conditions, even after adjusting for the strong influence of height, age, and BMI. According to these results, salivary concentrations of GM‐CSF were related with some PFTs variables putatively indicating the status of small airways and lung parenchyma, while glucagon concentrations were mainly associated with diminished airway resistances and less FVC. The potential influence of cytokines and hormones on PFTs is a novel concept that warrants more in‐depth investigation and characterization in children and adults.

## FUNDING INFORMATION

None.

## CONFLICT OF INTEREST STATEMENT

None.

## ETHICS STATEMENT

The protocol was approved by the institutional review board (approval number C07‐14), permission was obtained from scholastic authorities, and an informed consent letter and an assent letter were signed by parents and children, respectively.

## DECLARATION ON A RELATED MANUSCRIPT

A related paper reporting the raw and albumin‐adjusted concentrations of all salivary analytes derived from children included in the present study was published elsewhere (Chvez‐Alderete et al. Salivary concentrations of cytokines and other analytes in healthy children. Cytokine 2021:138:155379) and was mentioned in the second paragraph of Section 3 as reference (Chávez‐Alderete et al., 2021). Both papers do not overlap any meaningful data.

## Supporting information


Data S1.
