# Supplementary material for: Lung function is related to salivary cytokines and hormones in healthy children. An exploratory cross‐sectional study
Source: Physiol Rep. 2023 Dec 12;11(23):e15861. doi: 10.14814/phy2.15861 (PMC10716032; doi:10.14814/phy2.15861)
Supplement: Supplementary file 1 — Data S1. [file PHY2-11-e15861-s001.pdf]

SUPPLEMENTARY MATERIAL

Physiol Rep 2023

Lung Function is Related to Salivary Cytokines and Hormones in Healthy Children. An exploratory cross-sectional study

Laura Gochicoa-Rangel, PhD, Jaime Chávez, PhD, Rodrigo Del-Río-Hidalgo, MD, Selene Guerrero-Zúñiga, MSc, Uri Mora-Romero, MD, Rosaura Benítez-Pérez, MD, Luis Rodríguez-Moreno,MD, Luis Torre-Bouscoulet, MSc, and Mario H. Vargas, MSc

- **Supplemental Table S1** : Internal validation in males
- **Supplemental Table S2** : Internal validation in females
- **Supplementary Fig. S1**: Changes of standardized beta coefficients according to independent variables included in the regression models..

Supplemental Table S1. Internal validation of associations between pulmonary function tests and salivary analytes that remained in the final Model 1 of the multiple linear regression (male children, n=43)

Dependent variable (PFT, raw value)	Independent variable (p value adjusted by age, height, and zBMI)	Index	Estimate (original)	Training	Test	Optimism	Estimate (adjusted by optimism)
R5	GLP-1 (p=0.012)	R^2	0.6296	0.6532	0.5835	0.0697	0.5599
		g-index	0.1446	0.1440	0.1414	0.0026	0.1419
		Slope	1	1	0.9765	0.0235	0.9765
R5	Glucagon (p=0.003)	R^2	0.6531	0.6748	0.6073	0.0676	0.5855
		g-index	0.1473	0.1469	0.1442	0.0027	0.1446
		Slope	1	1	0.9780	0.0220	0.9780
R20	Glucagon (p=0.015)	R^2	0.5692	0.6003	0.5116	0.0888	0.4804
		g-index	0.0949	0.0953	0.0919	0.0034	0.0915
		Slope	1	1	0.9622	0.0378	0.9622
R5-R20	GM-CSF (p=0.012)	R^2	0.3962	0.4351	0.3114	0.1236	0.2725
		g-index	0.0606	0.0626	0.0578	0.0048	0.0558
		Slope	1	1	0.9309	0.0691	0.9309
X20	GM-CSF (p=0.007)	R^2	0.5564	0.5933	0.4928	0.1004	0.4559
		g-index	0.0287	0.0294	0.0278	0.0016	0.0271
		Slope	1	1	0.9545	0.0455	0.9545
Fres	IL-8 (p=0.046)	R^2	0.4408	0.4702	0.3586	0.1116	0.3291
		g-index	2.2564	2.2736	2.1367	0.1369	2.1195
		Slope	1	1	0.9480	0.0520	0.9480
Fres	GM-CSF (p=0.004)	R^2	0.5030	0.5371	0.4235	0.1136	0.3894
		g-index	2.4265	2.4869	2.3234	0.1635	2.2630
		Slope	1	1	0.9566	0.0434	0.9566
AX	GM-CSF (p=0.032)	R^2	0.5238	0.5629	0.4607	0.1021	0.4217
		g-index	0.5823	0.5935	0.5652	0.0283	0.5540
		Slope	1	1	0.9508	0.0492	0.9508
FEV_1	GM-CSF (p=0.047)	R^2	0.8255	0.8333	0.8026	0.0307	0.7948
		g-index	0.4372	0.4250	0.4329	-0.0078	0.4451
		Slope	1	1	0.9945	0.0055	0.9945
FEV_1/FVC	IL-8 (p=0.032)	R^2	0.4537	0.4814	0.3715	0.1099	0.3438
		g-index	3.8448	3.8361	3.6126	0.2235	3.6213
		Slope	1	1	0.9568	0.0432	0.9568
FEV_1/FVC	Adiponectin (p=0.014)	R^2	0.4645	0.4956	0.3880	0.1076	0.3569
		g-index	3.7819	3.7973	3.5773	0.2200	3.5619
		Slope	1	1	0.9636	0.0364	0.9636
DL_{CO}	IL-7 (p=0.026)	R^2	0.6451	0.6747	0.5918	0.0829	0.5622
		g-index	4.6864	4.7029	4.5797	0.1232	4.5633
		Slope	1	1	0.9619	0.0381	0.9619

For definition of abbreviations, see the main text.

Supplemental Table S2. Internal validation of associations between pulmonary function tests and salivary analytes that remained in the final Model 1 of the multiple linear regression (female children, n=53)

Dependent variable (PFT, raw value)	Independent variable (p value adjusted by age, height, and zBMI)	Index	Estimate (original)	Training	Test	Optimism	Estimate (adjusted by optimism)
R20	Insulin (p=0.027)	R^2	0.5621	0.6060	0.4950	0.1109	0.4511
		g-index	0.0983	0.0996	0.0943	0.0053	0.0930
		Slope	1	1	0.9424	0.0576	0.9424
R5-R20	Insulin (p=0.007)	R^2	0.4245	0.4728	0.3289	0.1438	0.2806
		g-index	0.0650	0.0664	0.0601	0.0063	0.0587
		Slope	1	1	0.9269	0.0731	0.9269
AX	IL-10 (p=0.04)	R^2	0.6005	0.6442	0.5431	0.1011	0.4994
		g-index	0.7941	0.8006	0.7651	0.0354	0.7587
		Slope	1	1	0.9594	0.0406	0.9594

For definition of abbreviations, see the main text.

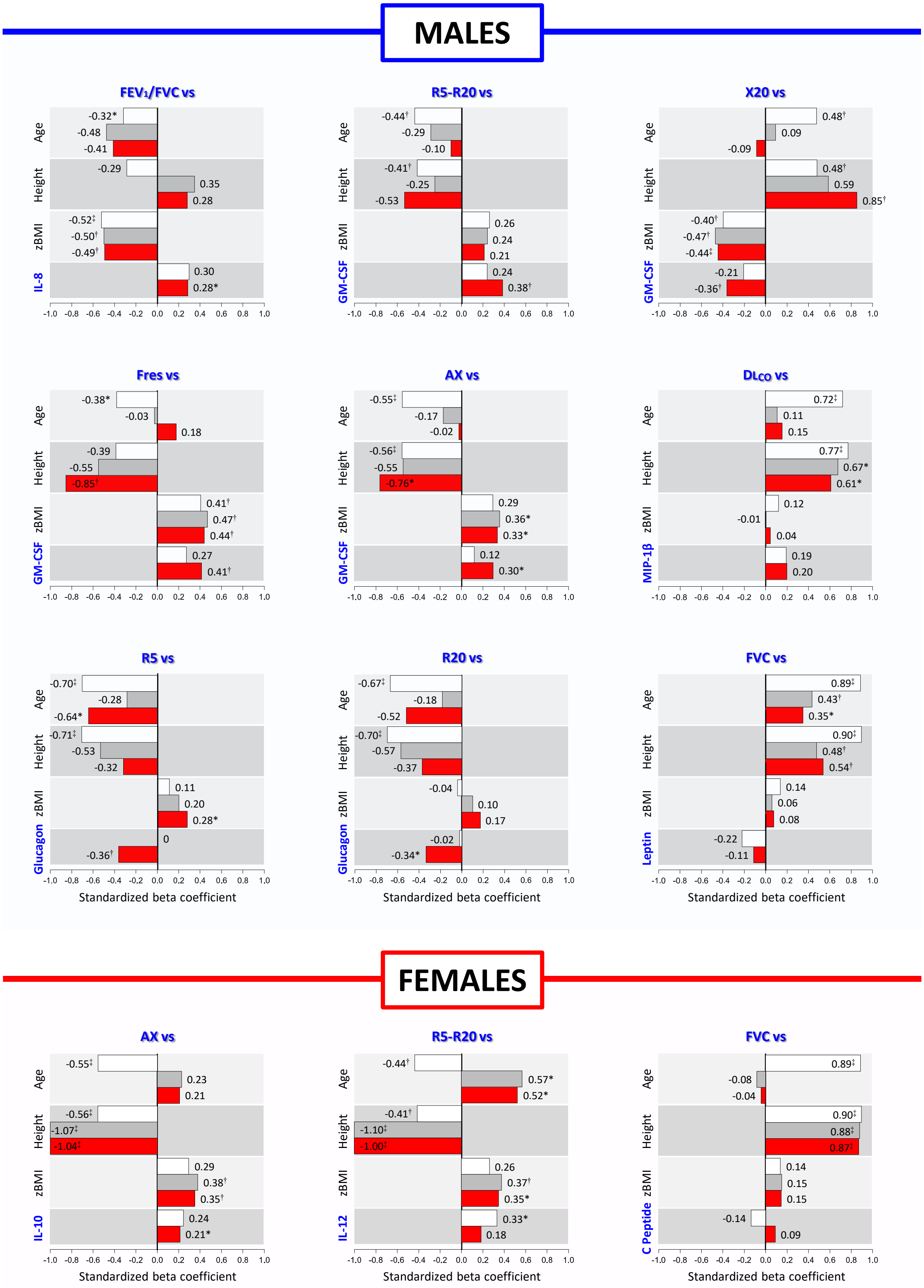

Supplementary Fig. S1. Changes of standardized beta coefficients according to independent variables included in the regression models. Standardized beta coefficients (bars) were obtained through liner regression and illustrate the relative contribution of independent variables for the prediction of lung function (dependent variable). **Open bars** = the regression model only included the variable indicated at the left (when only one independent variable is included, the standardized beta coefficient corresponds to the Pearson's correlation coefficient). **Gray bars** = the regression model included age, height, and z-score of body mass index for age (zBMI). **Red bars** = the regression model included age, height, zBMI, and the cytokine/hormone indicated at the left. $*p<0.05$, $\dagger p<0.01$, and $\ddagger p<0.001$.
